# Stem Cell-Based Therapeutic Approaches to Restore Sensorineural Hearing Loss in Mammals

**DOI:** 10.1155/2020/8829660

**Published:** 2020-08-01

**Authors:** Muhammad Waqas, Iram Us-Salam, Zainab Bibi, Yunfeng Wang, He Li, Zhongshou Zhu, Shuangba He

**Affiliations:** ^1^Department of Biotechnology, Federal Urdu University of Arts, Science and Technology, Gulshan-e-Iqbal Campus, Karachi, Pakistan; ^2^Department of Otolaryngology Head and Neck, Nanjing Tongren Hospital, School of Medicine, Southeast University, Nanjing 211102, China; ^3^ENT Institute and Otorhinolaryngology Department of Eye & ENT Hospital, Fudan University, Shanghai 200031, China; ^4^NHC Key Laboratory of Hearing Medicine (Fudan University), Shanghai 200031, China; ^5^Department of Otolaryngology, First Affiliated Hospital of Wenzhou Medical University, Wenzhou City, 325000 Zhejiang Province, China; ^6^Department of Otolaryngology, Ningde Municipal Hospital Affiliated of Fujian Medical University (Ningde Institute of Otolaryngology), Ningde, Fujian 352100, China

## Abstract

The hair cells that reside in the cochlear sensory epithelium are the fundamental sensory structures responsible for understanding the mechanical sound waves evoked in the environment. The intense damage to these sensory structures may result in permanent hearing loss. The present strategies to rehabilitate the hearing function include either hearing aids or cochlear implants that may recover the hearing capability of deaf patients to a limited extent. Therefore, much attention has been paid on developing regenerative therapies to regenerate/replace the lost hair cells to treat the damaged cochlear sensory epithelium. The stem cell therapy is a promising approach to develop the functional hair cells and neuronal cells from endogenous and exogenous stem cell pool to recover hearing loss. In this review, we specifically discuss the potential of different kinds of stem cells that hold the potential to restore sensorineural hearing loss in mammals and comprehensively explain the current therapeutic applications of stem cells in both the human and mouse inner ear to regenerate/replace the lost hair cells and spiral ganglion neurons.

## 1. Introduction

**Figure 1:**
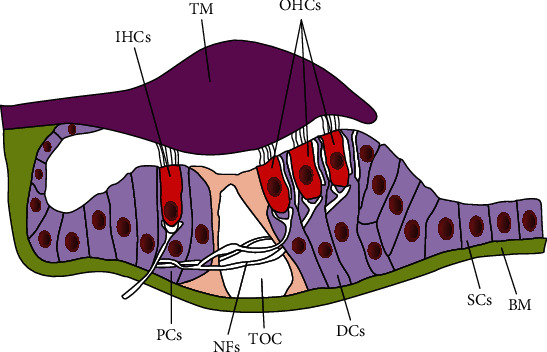
Schematic of the adult mammalian organ of Corti showing the normal arrangements of sensory and nonsensory cells on the basilar membrane. IHCs: inner hair cells; TM: tectorial membrane; OHCs: outer hair cells; PCs: pillar cells; NFs: nerve fibres; TOC: tunnel of Corti; DCs: Deiters' cells; SCs: supporting cells; BM: basilar membrane.

The inner ear is a sophisticated and sensitive sensory organ of the body. It contains three well-known functional structures: the cochlea for sound perception, the vestibule, and the semicircular canals for maintaining body equilibrium. The cochlea is responsible for understanding mechanical voices by transducing incoming sound vibrations into electrical impulses using hair cells (HCs) and then transmits these electrical impulses to the brainstem via spiral ganglion neuron cells (SGNs) [1–5]. The cochlear sensory epithelium has one row of inner hair cells (IHCs) and three rows of outer hair cells (OHCs) interdigitated with multiple layers of supporting cells (SCs) (Figure 1). The OHCs are responsible to amplify the sound vibrations while the IHCs manage to convert mechanical sound into neural signals that further transmit through SGN to the auditory circuit [6–10]. Due to the exquisite transducer in nature, sensitivity and the delicate structure of these cells make them a key target for the ototoxic damage. The three rows of OHCs that externally reside are more sensitive to HC damage as compared to the IHCs. Most of the hearing loss patients have the same pathological features in common such as the HC loss and the decreased number of SGNs [11, 12]. Ototoxic insult to the sensory HCs causes hair cell death, which is mainly due to the exposure to loud noise, use of aminoglycosides or chemotherapy regimens, viral infections, biological aging, and genetically inherited disorders [13–20]. The drug-induced damage also promotes the reduction of specialized synaptic structures between IHCs and SGNs followed by the later degeneration of SGN [21–23]. In order to regenerate the SGN, many different biomaterials have been applied to promote the neural stem cells to regenerate the SGNs [24–30]. Moreover, in recent years, many previous reports used transcription regulation, electrical stimulation, and magnetic regulation to promote the regeneration and maturation of SGNs [31–36].

One way to restore hearing loss is to produce new functional HCs to replace the lost HCs in the cochlea. Regeneration of HCs and SGNs after damage could possibly yield a treatment for sensorineural hearing loss [37, 38]. Stem cells have the potential to self-renew and the ability to differentiate into multiple cell types [39]. It is now well understood that a specific population of resident SCs marked with the stem cell markers Lgr5, Lgr6, Sox2, Sox9, Frizzled-9, EPCAM, and ABCG2 in the organ of Corti, commonly known as cochlear stem/progenitor cells, holds the stem cell-like potential to proliferate and differentiate to form both HCs and SCs [40–45]. However, the mammals only have very limited HC regeneration ability [42, 46–51]; thus, how to promote the HC regeneration ability and to promote the maturation of new regenerated HCs is the key scientific question in the hearing research field. Several research studies unravel the potential of different kinds of stem cells to generate HCs and SGNs, such as stem/progenitor cells, spiral ganglion-derived neural stem cells (endogenous stem cells), embryonic stem cells, and induced pluripotent stem cells (exogenous stem cells) [52–54]. In this review, we focus on the recent progress in the therapeutic use of different types of stem cells (endogenous and exogenous stem cells) to recover hearing function in the human and mouse inner ear.

## 2. Hearing Restoration Approaches

Hearing research science is primarily focused on formulating the best therapeutic strategies to renew the hearing cells (HCs and SGNs), thus restoring the natural hearing function and producing comfort to the millions of patients affected by this widely growing disorder [55]. Also, the damage to the HCs in the inner ear subsequently increases the risk of degeneration in the residual SGN. Therefore, it is essential to protect both the HCs and existing SGNs in the cochlea. More recently, stem cell therapy and gene therapy are the most promising therapeutic strategies to regenerate/replace HCs and SGNs in the cochlea after damage. Here, in this review, we will discuss the stem cell-based therapeutic strategies in the mammalian inner ear.

## 3. Characteristics of Stem Cells

Stem cells are better called the principal cells of the body that maintain their undifferentiated and unspecialized state in order to either directly transform into specialized cells or pursue the mitotic division to form new stem cells. Stem cells are used to restore cellular damage and recover the cell loss. Adult stem/progenitor cells, spiral ganglion-derived neural stem cells (SGN-NSCs), embryonic stem cells (ESCs), and inducible pluripotent stem cells (iPSCs) are kind of stem cells that are commonly used in therapeutics [56]. Adult stem/progenitor cells are inhabitants in the organ of Corti. There are various proteins like Lgr5, Lgr6, Sox2, Sox9, EPCAM, and ABCG2 that have been recognized as reliable cochlear stem/progenitor cell markers in the mouse and human inner ear [40, 57–61]. The molecular characterization and mechanism behind the higher proliferation and regeneration ability of Lgr5+, Lgr6+, and Sox2+ cochlear stem/progenitor cells have been thoroughly studied in mouse models using microarrays and RNA-Seq profiling, and a large dataset of genes has been identified that showed that multiple genes might regulate the proliferation and HC regeneration ability of stem/progenitor cells in the inner ear [48, 62–65]. Similarly, there is a proof for the existence of SGN stem cells that are commonly known as SGN-NSCs. These cells resided in the region of the SGN and are able to differentiate to form functional neurons [66, 67].

ESCs are pluripotent stem cells, obtained from the inner cell mass and hold the limitless potential to proliferate as well as to differentiate to form all three germ layers: ectoderm, mesoderm, and endoderm. Yet, the mouse ESCs have been more deeply investigated as compared with the human ESCs. ESCs have been widely used for in vitro culture system to deliberately induce the formation of different cell types such as liver cells, neuronal cells, cardiac cells, and pancreatic cells [68–73]. Thus, ESCs provide a significant resource of cells for replacement therapy in order to regenerate different tissues/organs.

The iPSCs are the adult differentiated cells that are genetically reprogrammed to form pluripotent stem cells. They hold a novel therapeutic ability to replace and repair the hearing cells (HCs and SGNs) in the inner ear. The mature skin fibroblast cells were the first reprogrammed iPSC generated by deliberately introducing the four crucial transcription factors including Klf4, Sox2, c-Myc, and Oct3/4 [74]. In therapeutics, the primary reason of iPSC generation from adult cells is to avoid the immunorejection in patients as the adult cells isolated, manipulated, and reintroduced as iPSCs in the same patient. Also, the use of iPSCs sufficiently decreased the ethical concerns about the use of stem cells as therapeutics.

## 4. Stem Cell Therapy in the Inner Ear

**Figure 2:**
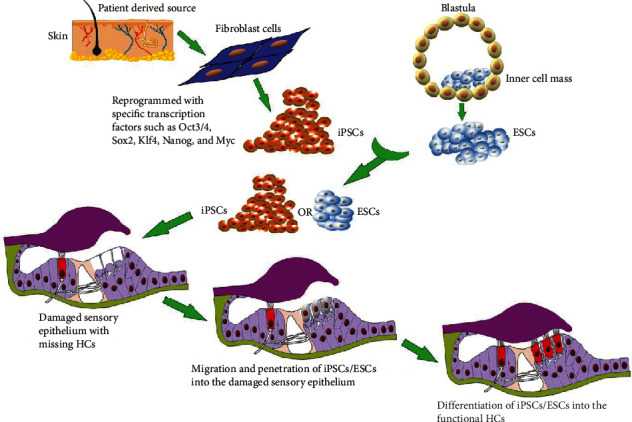
Schematic of the exogenous stem cell therapy showing the migration, penetration, and differentiation of embryonic stem cells (ESCs) or inducible pluripotent stem cells (iPSCs) to generate functional HCs in the damaged sensory epithelium.

The use of stem cell therapy in the inner ear is a promising approach to rescue the HC damage and to reestablish the hearing function. There are two possible stem cell-based approaches to treat deafness. The first is the restoration of existing stem cells in the inner ear by stimulating the resident stem cells within the organ of Corti, therefore allowing stem cells to replace the damaged HCs and rehabilitate the normal hearing mechanism. However, the basic difficulty with this approach is the insufficient number of resident stem cells in the inner ear that are not capable to restore hearing. The second is the exogenous supply of stem cells (stem cell transplantation) into the inner ear (Figure 2). This approach is implemented by either supplying stem cells into the scala tympani through the round window and triggering these cells to migrate into the cochlear sensory epithelium [75] or directly transplanting the stem cells into the scala media. However, the high concentration of potassium and the tight junction barriers make the endolymph environment very hostile for the survival of foreign stem cells [76]. Therefore, it is important to adopt the methods that create a more hospitable environment in the cochlea. There are few strategies to do so, such as replacing the scala media fluid with the more hostile media to stem cells, systemic administration of loop diuretic drug to lower the potassium concentration, and the use of sodium caprate that disrupts the tight junctions in the cochlea [77, 78].

### 4.1. Stem Cell-Based Therapeutic Approaches in the Human Inner Ear

The presence of endogenous stem/progenitor cells in the adult human sensory epithelium was evident, when the pure population of cells was marked with the stem cell marker ABCG2+ve isolated from dissociated human cochlear cells via flow cytometry. These dissociated human cochlear cells also form spheres in the in vitro culture system. However, the number of spheres generated in the experiments was inadequate to further characterize these spheres for the ability to regenerate functional HCs [61]. More recently, two prosensory markers EPCAM and CD271 have been used to separate the human fetal postmitotic HC progenitors. The 3D culture of EPCAM and CD271 marked cells in Matrigel allows the formation of cell colonies that displayed the expression of stem cell markers (Sox2, Sox9, and Fbxo2). These cells regain their proliferative capability and ultimately differentiate to form HC-like cells in vitro. However, the expression of Lgr5 was not observed in the cell colonies [60].

Multiple studies have attempted the transplantation of embryonic stem cells in the inner ear in order to regenerate HCs in vitro. There were few studies that reported on the use of human embryonic stem cells (hESCs) to differentiate to form HC-like cells. In one of the studies, the hESCs were triggered to differentiate under particular signals mandatory for the specification of the early otic placode and obtain otic progenitors that differentiate into HC-like cells displaying HC-specific marker, immature stereociliary bundles. These HC-like cells also showed the electrophysiological characteristics suggesting that they are functional HCs. Some other otic progenitors differentiate to form neuronal cells exhibiting specific neuronal markers and having electrophysiological properties, suggesting that these cells are also able to generate functional auditory neuronal cell fate [79]. The generation of human otic progenitors was relied on the fibroblast growth factor signaling, and the newly regenerated HC-like cells showed the specific HC markers and immature stereociliary bundles. However, these HCs are unable to build the fully matured HC cytoarchitecture, which is necessary to restore hearing function [80].

To overcome this nonfunctionality of matured HC-like cells, the three-dimensional culture system has been successfully used to generate the inner ear organoid from hESCs. The inner ear organoid has the genuine cytoarchitecture of HCs, SCs, and neuronal cells as expressive of the native inner ear sensory epithelium [81]. In vitro organoid culture system promotes the study of human inner ear development and presents a disease model for therapeutic research. The other researchers also followed this 3D culture system in their hESC experiments [82]. Although the exogenous ESC implantation is a promising strategy, one problem with the survival of implanted cells in the inner ear is the high concentration of potassium in the scala media. Lee et al. address this issue by preconditioning the scala media to reduce the potassium concentration before implanting the hESCs in the deaf guinea pig cochlea. Their results showed the increased survival of hESCs in the cochlea; however, some stem cells lose their pluripotency and differentiation ability as noted by the lower expression of the Oct3/4 marker [78]. The primary objective of this study is to figure out whether the hESCs survived after implantation in the animal model. The implanted hESCs showed attachment to the sensory epithelium even without full integration. Although there is a lack of clear evidence of integration, the application of sodium caprate strengthens the survival and encourages the differentiation of hESCs after implantation.

The capability to generate SGNs from stem cells is a compulsory requirement to develop stem cell therapy for SNHL. A group of researchers developed a protocol that allows the differentiation of hESCs into a pure population of otic neuronal progenitors (ONP) and SGN-like cells. Interestingly, the newly differentiated SGN-like cells express the specific SGN genotypic and phenotypic markers as well as extend their neurites towards the cochlear nucleus suggesting that the hESC-derived SGNs can closely replicate the features of functional human SGN [83]. Moreover, Hyakumura et al. recently described the use of human pluripotent stem cells (hPSCs) to derive sensory neuronal cells. They observed that the differentiated hPSC-derived neuronal cells formed synaptic connections with both the inner ear HCs and cochlear nucleus neurons in organotypic coculture. The contacts between hPSC-derived neuron cells and inner ear HCs and cochlear nucleus neurons are significantly positive for specific synaptic markers such as synapsin I and VGLUT1. This new auditory coculture model provides a clue for the use of stem cells in the bidirectional growth towards the target cells and tissue in the inner ear and brainstem [84]. However, the drawback is that the in vitro model does not provide enough clues to mimic in vivo physiological conditions. To address this issue, a new study demonstrated the use of nanofibrillar cellulose (NFC) hydrogel, which is a kind of artificial extracellular matrix (ECM). The use of NFC hydrogel together with the delivery of neurotrophic factor artificially creates a stem cell niche in in vitro and in vivo models. NFC hydrogel promotes the in vitro and in vivo survival and differentiation of hESC-derived ONP spheroids. The transplanted ONP spheroids have been shown to survive and neuronally differentiate into otic neuronal lineages both in vivo and in vitro. Interestingly, they also displayed protracted neurites towards the bony wall of the cochlea following the ninety days of transplantation [85].

There are some ethical concerns on the experimental utilization of human embryonic stem cells, and since then, much attention has been paid on the experimental generation of iPSCs from somatic cells to further transform to generate HC-like cells. Multiple strategies have been formulated successfully to first generate human iPSCs (hiPSCs) and then stepwise induce the differentiation of hiPSCs into the human inner ear HC-like cells [86]. There are different factors and signals that drive hiPSCs into otic sensory progenitor cells (OSPCs) to reestablish lost HCs. The rapid and efficient generation of OSPCs can be achieved by manipulating the cell signaling pathways such as modulation of Notch, Wnt, FGF, and TGF-*β* through the use of the differentiated monolayer culture system [87, 88]. These efficiently generated OSPCs could be established and used for disease modeling and cell-based therapies.

The correction of gene mutation in iPSCs stimulated from somatic cells of diseased persons is a promising way to treat hereditary SNHL. In two different studies, researchers begin iPSC formation from a deaf patient carrying Myo7a and Myo15 mutations that are mainly responsible for deafness. CRISPR/CAS 9 gene-editing tool is used to genetically rectify the Myo7a and Myo15 mutations and observe that the HC-like cells derived from the corrected iPSCs exhibited the recovered organization of the stereociliary-like structures and complete morphological and functional restoration of HCs [89, 90]. Moreover, in another study, the iPSCs were generated from the fibroblast cells of a MERRF syndrome patient with A8344G mutation of mitochondrial DNA. The iPSCs were driven by a set of transcription factors Atoh1/Rfx1/Rfx3 that significantly increased the differentiation ability of iPSCs into Myo7a+ve cells. These newly differentiated HC-like cells displayed the expression of HC-related genes and facilitated the HC-like cells with more mature stereociliary bundles [91]. Also, a recent study reported that the reprogramming of urinary cells isolated from the healthy human individual turns them into iPSC. These iPSCs were further differentiated to form otic epithelial and HC-like cells. There were two different observations recorded in vivo and in vitro. In vitro conditions displayed that the newly reprogrammed HC-like cells appear to be completely mimicked in morphological and electrophysiological characteristics as with the normal HCs. However, in vivo conditions showed that a very limited number of transplanted HC-like cells moved and integrated into the resident site of original HC and fewer cells formed neuronal connections with SGNs [92].

One main concern regarding the use of iPSCs is their genetic integrity, as the use of viral vectors during reprogramming of these cells might cause the insertional mutagenesis. To address this query, Boddy et al. proposed a nonintegrating mRNA-based reprogramming of human-induced pluripotent stem cell (hiPSC) lines. The integration-free hiPSC lines were allowed to culture in the presence of FGF3 and FGF10 that trigger the process of hiPSC line differentiation into otic progenitors as confirmed by the detection of otic markers Pax2, Pax8, Sox2, and Foxg1. Subsequently, the purified otic epithelial and neuroprogenitors were differentiated to generate HC-like cells and neurons [93].

In addition, the iPSCs also served as a resource for the replacement therapy of neurons in the damaged cochlea. A study demonstrated that the hiPSC-derived neurons innervate with the developing HCs and form presynaptic connections in the in vitro coculture system. Those hiPSC-derived neural progenitors cocultured with HCs at an earlier stage of differentiation displayed a higher innervation potential as compared to the other neural progenitors [94]. However, the transplantation of these neural progenitors in the damaged cochlea remains a challenge. A recent work explained the specific stepwise neural induction method for hiPSCs to eliminate the undifferentiated cells from neurons. The hiPSC-derived neural progenitors were first established on Matrigel. Then, these neural progenitors differentiated into neurons on a 3D collagen matrix. Lastly, the hiPSC-derived neurons cultured on a 3D collagen matrix were transplanted into the guinea pig cochlea [95]. The results showed that hiPSC-derived neuronal cells expressed specific neuronal markers and the survival of transplant-derived neurons can be achieved by controlling the inflammatory response.

### 4.2. Stem Cell-Based Therapeutic Approaches in the Mouse Inner Ear

The embryonic stem cells derived from mice were first used in an experiment to produce HC-like cells in vitro by formulating a proper stepwise differentiation strategy. These differentiated HC-like cells showed the full expression of HC-specific markers observed via gene expression profiling and immunostaining [96]. The *Barhl1* is a deafness gene expressed in the developing hair cells. It plays an important role in the differentiation of mouse embryonic stem cells (mESCs) into HC-like cells. The targeted disruption of *Barhl1* hindered the differentiation of mESC-derived HC-like cells in vitro [97]. Moreover, the use of mouse pluripotent stem cells displayed the successful in vitro differentiation of both embryonic stem cells and iPSCs into the HC-like cells. These newly differentiated HC-like cells were generated by applying the scheme to mimic the basic concepts of early embryonic and normal otic development. In the in vitro feeder layer of the chicken utricle, stromal cells were used for differentiation and maturation of these embryonic and iPSCs into the HC-like cells. The newly formed cells showed that the mechanosensing stereociliary structures on their surfaces resemble the mouse vestibular HCs and were responsive to the mechanical stimulation [52]. On account of the earlier detailed report, the multiple strategies are formulated based on the use of the feeder cell layers. One study on this aspect reported that the application of the feeder cell layer (ST2 stromal cell-conditioned medium) together with the transfection of the Atoh1 transcription factor in mouse embryonic stem cells efficiently induces the formation of HC-like cells in vitro [98].

Despite the use of the feeder cell layer, some other strategies such as three-dimensional (3D) cultural systems have also been used to transform mESCs into HC-like cells, SCs, and neuronal cells. The advantage of the 3D cultural system is that the neuronal cells established synaptic connectivity with the HCs. Furthermore, the aggregate of mESCs in the 3D culture system has been guided to mimic the normal development by sequentially generating the nonneural ectoderm expressing multiple marker genes (including FOXI3, GATA3, DLX5, SIX1, and EYA1), preplacodal ectoderm, and otic placode (expressing PAX2 and PAX8 genes) [99–101]. Also, the Wnt activation enhances the inner ear organoid development from mESCs in the 3D culture system [102]. Moreover, a recent study defines the new protocol to derive inner ear organoids from mutant mESCs under chemically defined conditions. In this protocol, they developed the 3D culture method to generate the inner ear organoid from mESCs, which differentiate to form the functional HCs and innervated by the sensory-like neuronal cells. In this approach, firstly, the mESCs were derived from the blastocyst stage of a Pax2 fluorescent reporter mouse line. Then, these Pax2^EGFP/+^ cells were used for inner ear organoid formation to understand the otic induction. The results displayed the higher expression of Pax2 and active stimulation of ERK downstream of the FGF signaling pathway in inner ear organoid development. The expression of Pax2 was persistent throughout the formation of sensory HCs, and the cochlear neurons established synaptic connections with HCs in the organoids [103, 104].

In addition, there is a novel exploration of transcriptional machinery that controls the HC fate and differentiation of mESCs. The simultaneous overexpression of three transcription factors, *Gfi1*, *POU4f3*, and *Atoh1*, directly stimulates the genetic programming in mESCs that leads to the sensory HC generation in vitro. The newly generated HCs express various HC-specific markers and revealed the polarized membrane protrusions on the HC surfaces similar to the stereociliary bundles [105]. The differentiation of mESCs by deliberate induction in culture leads to the migration of progenitor cells derived from mESCs into the cochlea. These cells also expressed the specific HC markers after transplantation into the inner ear [106, 107]. Also, it is a prerequisite condition to integrate the mESC-derived neurons into the central nervous system (CNS) for functional synaptic connectivity. An in vitro coculture system has been developed in which the mESCs were first induced to form mESC-derived SGN-like cells, and then, these SGN-like cells were allowed to coculture with CN neurons for 4-6 days in the presence of thrombospondin-1. The results showed the development of neural connections between mESC-derived SGN-like cells and CNS as confirmed by the expression of pre- and postsynaptic markers on the newly formed synaptic structures [108]. In contrast to mESCs, the use of mouse iPSCs to generate HCs and SGNs is not promising at all yet. Multiple studies reported that the mouse iPSCs could differentiate to form HC-like cells and SGNs after transplantation into the mouse cochlea; however, there is no significant improvement observed in the threshold of auditory brain response (ABR) [109–111].

Until now, in vitro studies regarding both human and mouse ESCs and iPSCs demonstrated that the specific culture conditions allow the stem cells to differentiate and achieve the desired cell fate such as HC-like cells and SGNs [112, 113]. The introduction of stem cell-derived progenitors at the spot of injury in the inner ear permits the transplanted cells to integrate and express the HC markers in the cochlear and vestibular sensory epithelium in vivo [96]. However, a very limited number of studies examined the assimilation of newly differentiated HCs into the mammalian inner ear. In multiple studies, the results regarding the implantation of stem cells to generate the functional HCs at the location of the damaged mammalian inner ear are uncertain [114, 115]. There is a limited number of transplanted cells that converted to form the required cell fate such as HCs, SCs, and neuronal and glial cells while a large number of cells were unable to achieve relevant cell types even after several weeks of transplant. The possible reason for this uncertainty is the change in the in vivo microenvironment in the mammalian cochlea, which is absolutely different from the in vitro culture conditions where HCs were generated. Another complexity is correctly targeting the damaged cochlear regions where the HCs are actually required and generation of adequate functional HCs at those sites. Also, another considerable challenge with the stem cell therapy for HC regeneration is the appropriate HC, SC, and SGN integration and orientation within the specific sites in the cochlea.

## 5. Conclusion

Hearing research is mainly focused on developing different strategies to design therapeutics that help to initiate the HC regeneration/replacement process in the inner ear to ultimately recover hearing loss. Multiple studies on the animal model have been successfully conducted that allow the clinical applications of endogenous and exogenous stem cells in order to regenerate/replace HCs in the mammalian inner ear. However, there are numerous challenging questions that need to be dealt with before executing these therapeutic strategies in humans. Some of them include the risk of tumorigenesis after implanting the stem cells, possible detrimental effects to the patients, and appropriate and controlled growth of stem cells at the site of cell transplantation in the cochlea. Also, the high cost of stem cell therapy makes it unreachable for a large number of hearing loss patients. In addition, the success rate of stem cell therapy is not high enough yet. Regardless of these limitations, stem cell therapy is still a promising future strategy to start HC regeneration/replacement in the adult mammalian cochlea to recover sensorineural hearing loss.

## Data Availability

This is a review article, and the data supporting this review article are from previously reported studies and datasets, which have been cited in the text.
